# The First Reported Case of Majocchi's Granuloma with* Malbranchea *sp. in an Immunocompetent Patient

**DOI:** 10.1155/2017/9196528

**Published:** 2017-08-30

**Authors:** Anusha Govind, Nathan L'Etoile, Gustavo Vasquez

**Affiliations:** ^1^Thomas Jefferson University Hospital, 1015 Chestnut St., Suite 1020, Philadelphia, PA 19107, USA; ^2^Sidney Kimmel College of Medicine, 1025 Walnut St., Suite 116, Philadelphia, PA 19107, USA

## Abstract

Majocchi's granuloma is a rare condition in which a dermatophyte invades the deeper layers of the dermis and subcutaneous tissue and can often be misidentified and treated as eczema. It has a variable presentation ranging from cutaneous lesions to deeper infections in immunocompromised patients. No prior cases have described the formation of Majocchi's granuloma with the deuteromycetes,* Malabranchea*.

## 1. Introduction 

Majocchi's granuloma has been described in the literature in both immunocompetent and immunocompromised patients. The organisms responsible for this infection are typically identified as keratinophilic dermatophytes. We have described the first case of Majocchi's granuloma as a result of* Malbranchea *species.

## 2. Case

**Figure 1:**
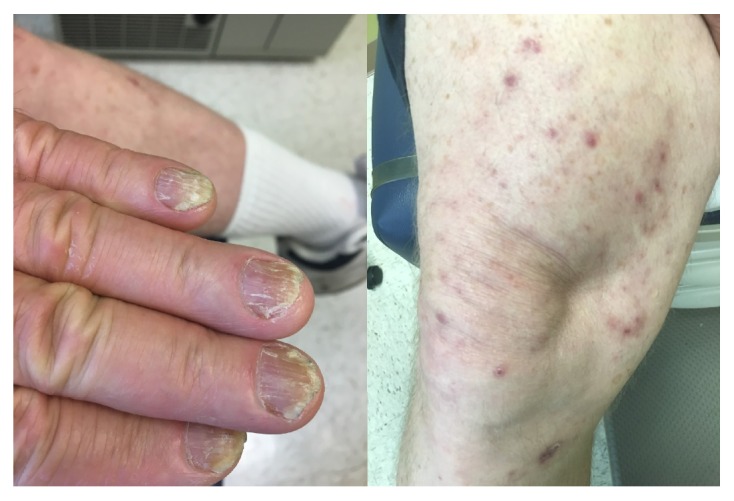
Dystrophic nail findings and circular purple papules on patient's lower extremity prior to therapy.

A 65-year-old male with history of non-insulin-dependent diabetes mellitus (HbA1c of 6.5%) and schizoaffective disorder presented with multiple new skin lesions starting in July 2016. The patient had underlying eczema and he complained that his lesions were worsening over the prior three weeks, starting on his right knee, with subsequent spread to his left knee and bilateral upper extremities. The lesions appeared different from his baseline eczema and did not itch. The patient's primary care physician noticed this rash on the entire circumference of his upper extremities and extensor surface of his thighs bilaterally (Figure 1). The lesions were described as purple or dark red plaques and papules with a discrete circular pattern, without underlying erythema. In addition, some of the papules had minimal pustular drainage. There were fewer lesions in sun exposed areas; the patient was started on triamcinolone cream for presumed guttate psoriasis. However, he showed no improvement at follow-up three weeks later.

The patient reported no sick contacts or international travel. The only travel he reported was to Montana in April of 2016 and New Mexico in 2015, but he did not have any acute illnesses while traveling or subsequently. The patient lives with his significant other and does not have any pets at home. He denied any known insect bites.

**Figure 2:**
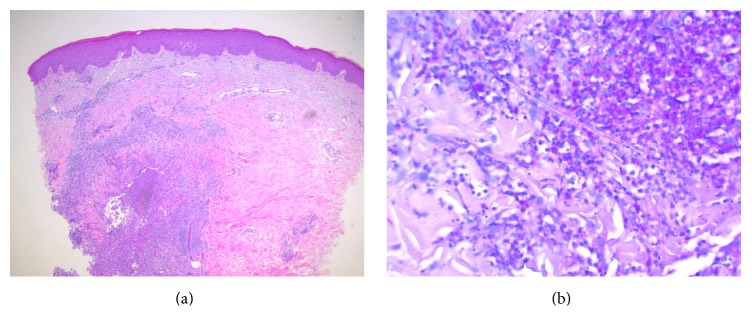
(a) Haematoxylin and eosin stain demonstrating granuloma formation. (b) Periodic acid-Schiff stain demonstrating fungal hyphae.

The differential diagnosis included atypical erythema migrans, viral exanthem, tinea, or potentially Majocchi's granuloma (MG). A punch biopsy of the left medial forearm and left thigh lesion demonstrated a deep fungal infection and dense suppurative and granulomatous inflammation in the dermis centered around a follicle (Figure 2(a)), with budding yeast and hyphal forms within the follicle (Figure 2(b)), which was consistent with Majocchi's granuloma. PAS stain noted budding yeast forms, and Gram, AFB, and Fite stains were unrevealing. A deep fungal culture grew a very light growth of* Malbranchea *species, identified by morphology, with no evidence of histoplasmosis or blastomycosis. The patient was evaluated for immunosuppressive conditions and tested negative for HIV, hepatitis B, and hepatitis C. Complete blood count and metabolic panel were normal.

The patient was prescribed itraconazole but could not obtain it secondary to financial reasons. Therefore, he was started on oral terbinafine for Majocchi's granuloma, and lesions started to resolve. Subsequent fungal blood cultures were negative.

## 3. Discussion 

Majocchi's granuloma (also known as nodular granulomatous perifolliculitis) is a rare condition in which a dermatophyte invades the deeper layers of the dermis and subcutaneous tissue. The condition typically presents as follicular or perifollicular papular or pustular lesions on the extremities in immunocompetent patients. In immunocompromised patients, it can present as deeper plaques and subcutaneous lesions. Majocchi's granulomas generally start at the site of hair follicles and frequent trauma (e.g., shaving) and appear as a firm papule in immunocompetent patients. Repetitive trauma allows for the introduction of keratinophilic dermatophytes to destroy keratinocytes and propagate a deeper infection [1]. In immunocompromised patients, however, these lesions occur most commonly on the forearms or legs and progress to form perifollicular pustules or fluctuant abscesses. Deep infections resulting in abscesses and ulceration are normally restricted to immunocompromised patients; however, cases of ulceration have been reported in immunocompetent individuals [2]. Additionally, similar to the patient presented here, one case of deep infection was misdiagnosed and treated as eczema [3]. In a review by Ilkit et al., 12.7% of patients with Majocchi's granuloma reported prior topical corticosteroid use [1, 3]. Although MG is typically described as a dermatophytic invasion, other causative agents have rarely been described including* Aspergillus *and species from the* Phoma* genera [1].

*Malbranchea *is a saprotrophic and keratinophilic deuteromycetes commonly found in soil and as well as dust samples in houses and hospitals in India.* Malbranchea cinnamomea* has been found in agricultural environments in United States [4, 5]. It has been described as a causative agent for dystrophic nails, especially in patients with underlying chronic illnesses. Of 245 patients evaluated for saprophytic infections of the nails and skin,* Malbranchea *was isolated from skin in 1% of the patients [6]. There are very few reports in the literature illustrating the infectious potential of* Malbranchea*.* Malbranchea pulchella *was described as an agent for chronic sinusitis in a patient in 1994 [7]. A study published in 2015 evaluated various opportunistic mold pulmonary infections in patients with tuberculosis and* Malbranchea saccardo* was a coexisting pathogen in 0.3% cases [7, 8]. No cases of Majocchi's granuloma have been identified with growth of* Malbranchea* species to date.

Majocchi's granuloma is important to consider in the differential diagnosis of both immunocompetent and immunocompromised patients with eczema not responding to standard therapy.
